# Solvothermal Preparation and Electrochemical Characterization of Cubic ZrO_2_ Nanoparticles/Highly Reduced Graphene (HRG) based Nanocomposites

**DOI:** 10.3390/ma12050711

**Published:** 2019-02-28

**Authors:** Mohammed Rafi Shaik, Manawwer Alam, Syed Farooq Adil, Mufsir Kuniyil, Abdulrahman Al-Warthan, Mohammed Rafiq H Siddiqui, Muhammad Nawaz Tahir, Joselito P. Labis, Mujeeb Khan

**Affiliations:** 1 Department of Chemistry, College of Science, King Saud University, P.O. 2455, Riyadh 11451, Saudi Arabia; rafiskm@gmail.com (M.R.S.); malamiitd@gmail.com (M.A.); sfadil@ksu.edu.sa (S.F.A.); mufsir@gmail.com (M.K.); awarthan@ksu.edu.sa (A.A.-W.); rafiqs@ksu.edu.sa (M.R.H.S.); 2 Chemistry Department, King Fahd University of Petroleum and Materials, Dhahran 31261, Saudi Arabia; muhammad.tahir@kfupm.edu.sa; 3 King Abdullah Institute for Nanotechnology, King Saud University, Riyadh 11451, Saudi Arabia; jlabis@ksu.edu.sa

**Keywords:** ZrO_2_ nanoparticles, graphene nanocomposites, solvothermal synthesis, electrochemical studies

## Abstract

A single-step solvothermal approach to prepare stabilized cubic zirconia (ZrO_2_) nanoparticles (NPs) and highly reduced graphene oxide (HRG) and ZrO_2_ nanocomposite (HRG@ZrO_2_) using benzyl alcohol as a solvent and stabilizing ligand is presented. The as-prepared ZrO_2_ NPs and the HRG@ZrO_2_ nanocomposite were characterized using transmission electron microscopy (TEM) and X-ray diffraction (XRD), which confirmed the formation of ultra-small, cubic phase ZrO_2_ NPs with particle sizes of ~2 nm in both reactions. Slight variation of reaction conditions, including temperature and amount of benzyl alcohol, significantly affected the size of resulting NPs. The presence of benzyl alcohol as a stabilizing agent on the surface of ZrO_2_ NPs was confirmed using various techniques such as ultraviolet-visible (UV-vis), Fourier-transform infrared (FT-IR), Raman and XPS spectroscopies and thermogravimetric analysis (TGA). Furthermore, a comparative electrochemical study of both as-prepared ZrO_2_ NPs and the HRG@ZrO_2_ nanocomposites is reported. The HRG@ZrO_2_ nanocomposite confirms electronic interactions between ZrO_2_ and HRG when compared their electrochemical studies with pure ZrO_2_ and HRG using cyclic voltammetry (CV).

## 1. Introduction

Metal oxide nanoparticles (NPs) possess excellent electrochemical properties and have long been applied in several electrochemical applications including electrochemical sensors, energy conversion and energy storage [1,2]. Particularly, transition metal oxides NPs have received considerable attention because of their redox properties and ease of large-scale synthesis [3,4,5]. Additionally, it is relatively easy to tune the structural properties of these NPs, such as size, morphology and crystallinity, which allows systematic investigation of the structure-electrochemical property relationship [6,7]. Recently, the demand for the development of different electrochemical sensors for the detection of various analytes, especially biomolecules, has significantly increased in the medical field to monitor changes in the concentrations of biochemicals in the human body [8].

In this regard, a denotative interest has been generated in employing various metal oxide NPs as electrode materials due to their remarkable properties, such as good conductivity, relative chemical inertness etc. [9,10,11]. However, several metal oxide NPs with promising chemical and thermal stability have not yet been properly explored. Among these, ZrO_2_ NPs, with excellent chemical inertness and low toxicity, could be a candidate to display potential electrochemical properties. They have been applied as an ideal electrode material in biosensors [12,13]. They are a P-type semiconductor with plenty of oxygen vacancies on the surface, and they possess high ion-exchange capacity and redox activities, making them useful in many electrochemical processes [14]. Moreover, due to their high mechanical strength and excellent optical and thermal properties, ZrO_2_ NPs have also been applied in various other fields.

In particular, cubic ZrO_2_ exhibits decent electrochemical properties due to its excellent electrical and surface charge properties, high mechanical strength, superior chemical and thermal stability and biocompatibility [15]. Therefore, it has gained significant prominence as a transducer in the fabrication of chemicals and biosensors [16]. However, electrochemical applications of ZrO_2_ have been seriously hindered due to its moderate redox behavior and low electrochemical active surface area [17]. Materials with high surface areas and good electrical conductivity can be potentially applied to improve the electrochemical properties of ZrO_2_. In this regard, graphene has attracted tremendous attention as a substrate material, as it possesses excellent electrochemical conductivity due to high mechanical flexibility, good stability and rapid heterogeneous electron transport (HET) properties, which are often required for the production of effective chemical and biosensors [18,19,20]. 

Therefore, the development of facile and low-cost methods for the large-scale preparation of highly crystalline, monodispersed ZrO_2_ NPs and graphene-based ZrO_2_ nanocomposites (HRG@ZrO_2_) remains an area of interest for researchers. Various methods have been explored for the preparation of stabilized ZrO_2_ NPs and HRG@ZrO_2_, including electrochemical, hydrothermal and solvothermal methods etc. [13,21,22]. For instance, ZrO_2_ was homogeneously distributed on a graphene oxide support using an in-situ electrochemical redox reaction between zirconyl chloride and graphene oxide [13]. Teymourian et al. prepared HRG@ZrO_2_ nanocomposites by a hydrothermal method at 180 °C for 18 h in which the colloidal suspension of graphene oxide, ZrOCl_2_ solution and hydrazine monohydrate was used [23]. Similarly, various other methods have also been reported [24].

In most studies, the as-prepared HRG@ZrO_2_ nanocomposites have demonstrated excellent electrochemical properties and were applied as efficient chemical and/or biosensors. However, studies on the changes in the electrochemical properties of ZrO_2_ NPs after the inclusion of graphene are rarely reported. The size of the NPs and surface chemistry play important roles in defining the electrochemical properties of materials [25]. Particularly, the size of NPs is an important factor in defining their electronic and optical properties. For instance, unusual quantization and luminescence effects have been observed in ultra-small (1–10 nm) TiO_2_ NPs [26]. The control of size and morphology of nanomaterials mostly depends on the synthetic methodology, and methods which offer good control over these parameters under facile conditions (low temperature) are highly desirable [27]. Therefore, the synthesis of ultra-small ZrO_2_ NPs and HRG@ZrO_2_ nanocomposites and their comparative electrochemical study may provide valuable information. For this purpose, a synthetic protocol is required which employs the same conditions to synthesize both materials. In our previous study, we reported the synthesis of HRG@ZrO_2_ nanocomposites using a facile and one-step solvothermal strategy using benzyl alcohol as solvent [28]. However, benzyl alcohol is known to exhibit a significant effect on the size of the resulting metal oxide NPs by slight variation in the reaction conditions, including temperature and concentration of benzyl alcohol [27]. 

**Scheme 1:**
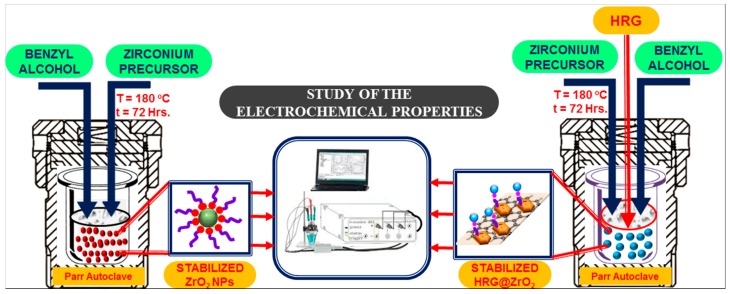
Graphical representation of chemically synthesized ZrO_2_ NPs and evaluation of their electrochemical properties.

Therefore, here we present a slightly modified method to prepare the smaller size ZrO_2_ NPs and HRG@ZrO_2_ nanocomposites under similar set of conditions (cf. Scheme 1). In this method, benzyl alcohol was used as a solvent, which also functioned as stabilizing ligand and facilitated the homogeneous distribution of smaller size ZrO_2_ NPs (~2 nm) on the surface of graphene nanosheets. Full characterization of HRG@ZrO_2_ nanocomposite was reported in our earlier study, while the as-prepared ZrO_2_ NPs are characterized by various techniques, such as, XRD, UV-vis, and FT-IR spectroscopies, and HR-TEM. Furthermore, the electrochemical characteristics of the as-prepared ZrO_2_ NPs and previously prepared HRG@ZrO_2_ nanocomposites are also investigated and compared. 

## 2. Materials and Methods

### 2.1. Materials

Zirconium (IV) isopropoxide isopropanol complex (Zr(OCH(CH_3_)_2_)_4_·(CH_3_)_2_CHOH) (99.9%), benzyl alcohol (99.0%), hydrazine hydrate (reagent grade, N_2_H_4_ 50–60%), KMnO_4_ (99%), H_2_O_2_ (30 wt%), H_2_SO_4_ (98%), NaNO_3_ (99%) and solvents were obtained from Sigma-Aldrich. 

### 2.2. Preparation of HRG@ZrO_2_ and ZrO_2_ NPs

The ZrO_2_ NPs and HRG@ZrO_2_ nanocomposite was prepared using our previously reported method [28] with slight modification. In order to alter the size of NPs, the temperature and amount of benzyl alcohol was slightly varied. For the preparation of ZrO_2_ NPs, 1.25 g of zirconium complex was added into 30 mL of benzyl alcohol in a Teflon cup. The resulting mixture was vigorously stirred to completely dissolve the whole zirconium complex. The Teflon cup was fixed into a 50 mL autoclave (stainless steel) and heated to 180 °C. The reaction was stopped after 3 days (72 h) and the vessel was cooled down to obtain a turbid suspension (white). The resulting product was separated as a white crystalline powder by centrifugation. Subsequently, the product was washed with tetrahydrofuran (THF) and dried in an oven at 70 °C to obtain ultra-small ZrO_2_ NPs, i.e., with size range of 1–2 nm. In order to prepare HRG@ZrO_2_ nanocomposite, separately prepared HRG (double the amount of zirconium complex) was added into benzyl alcohol. HRG-BA was prepared by dispersing 100 mg of HRG in 30 mL of benzyl alcohol by sonication for 30 min. The resultant mixture was transferred into a Teflon cup which was inserted into a 50 mL stainless steel autoclave and heated to 180 °C. The HRG-BA was also isolated in a similar manner as HRG@ZrO_2_ nanocomposite was obtained.

### 2.3. Characterization

Samples of ZrO_2_ NPs obtained by solvothermal synthesis with zirconium isopropoxide in benzyl alcohol were investigated by HRTEM (HRTEM using a JEOL JEM-2100F (Tokyo, Japan) instrument (The accelerating voltage used for TEM measurements is 200 kV). XRD measurements were performed on a D2 Phaser X-ray diffractometer (Bruker, Karlsruhe, Germany) with Cu Kα radiation (λ = 1.5418 Å). Furthermore, FT-IR spectra were measured on a Perkin Elmer, Spectrum 100 FT-IR spectrometer (FT-IR, Perkin Elmer, Waltham, MA, USA). To exclude the possibility of unbound benzyl alcohol molecules contaminating the surface of ZrO_2_ NPs, the resulting product was redispersed in DI water via sonication for several minutes (30 min). Thereafter, the sample was isolated by centrifugation at high speed (9000 rpm) for several minutes. The process was repeated twice to obtain a pure ZrO_2_ sample.

Meanwhile, a PerkinElmer lambda 35 (USA) UV-vis spectrophotometer (PerkinElmer lambda, Waltham, MA, USA) was used for the optical measurements. Thermal analysis was carried out on a TGA/DSC1(Mettler Toledo AG, Analytical, Schwerzenbach, Switzerland). The TGA measurements were performed under nitrogen at a heating rate of 10 °C/min. Electrochemical measurements were recorded on Autolab Potentiostat/galvanostat, PGSTAT 204-FRA32 control with NOVA software (Metrohom Autolab B.V.Kanaalweg 29-G, 3526 KM Utrecht, The Netherlands) in three electrode system at room temperature in 5 M KOH. The working electrodes used were ZrO_2_ and HRG@ZrO_2_ modified glassy carbon electrodes, while was used platinum as a counter electrode, and Ag/AgCl was used as a reference electrode. Prior to coating, a slurry of materials was prepared (70% ZrO_2_/HRG@ZrO_2_, 30% ethyl cellulose) in 5 M KOH and coated on the surface of Glassy carbon electrode (GCE). Subsequently, the electrode was dried at 60 °C for 2 h to get a homogenous and dried layer over electrode surface. The current response was measured from −1.2 to 1.2 V. X-ray photoelectron spectroscopy. XPS spectra were measured on a PHI 5600 Multi-Technique XPS (XPS, Physical Electronics, Lake Drive East, Chanhassen, MN, USA) using monochromatized Al Kα at 1486.6 eV. Atomic concentrations were calculated using MULTIPAK 9.4.1.2 software. Peak fitting was performed with CASA XPS Version 2.3.14 software. Raman spectroscopy was performed using Jobin Yvon LabRAM HR800 (HORIBA FRANCE SAS, Les Ulis, France) confocal Raman system equipped with an optical microscope Olympus BX41 and Peltier-cooled CCD detector, 633 nm He-Ne laser.

## 3. Results and Discussion

### 3.1. XRD Analysis

**Figure 1:**
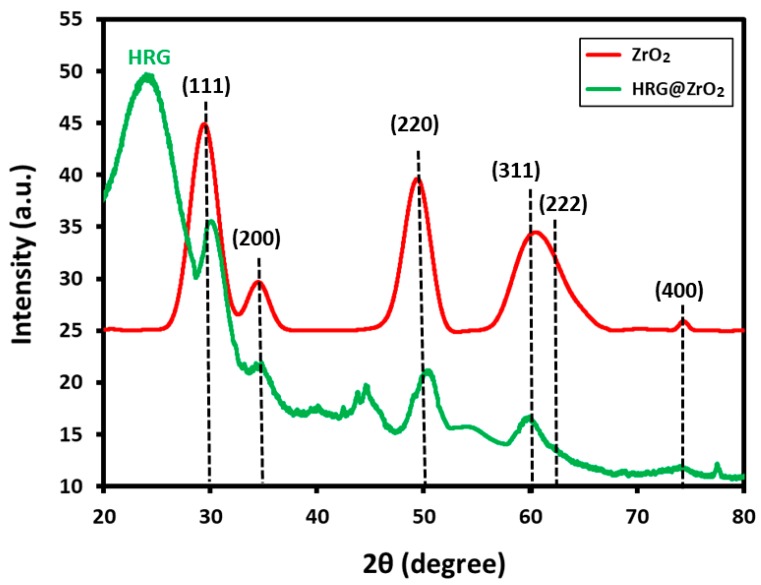
XRD spectrum of pure ZrO_2_ NPs and HRG@ZrO_2_ nanocomposite depicting the cubic phase of ZrO_2_ in both samples.

Benzyl alcohol is an excellent agent, which can be applied in a general route to prepare nanosized, low-dimensional transition metal oxides, including ZrO_2_ NPS. It provides an unprecedentedly versatile reaction system for the preparation of spherical, “quasi” zero-dimensional nanoparticles [27]. Moreover, appropriate thermal conditions and relative amounts of benzyl alcohol and metal precursor allow excellent control over particle size, phase, and the crystallinity of the resultant material. In our previous report, HRG@ZrO_2_ prepared at 210 °C has rendered ~3–4 nm-sized ZrO_2_ NPs on the surface of HRG, while in this study, slight variations of temperature from 210 °C to 180 °C led to the formation of ultra-small ZrO_2_ NPs (~2 nm), both in the case of the pure ZrO_2_ sample and HRG@ZrO_2_ nanocomposite. The formation and crystallinity of ZrO_2_ NPs was initially confirmed by XRD analysis. Figure 1 shows the XRD spectrum of ZrO_2_ NPs, which clearly confirms the presence of the cubic phase. All the diffraction peaks can be assigned to the pure cubic phase of ZrO_2_. The peaks are perfectly matched with the reported data (JCPDS No. 27–0997) [29]. The absence of any additional peaks in the spectrum clearly indicates that the sample contains a pure cubic structure. Five prominent peaks observed at 2*θ* values of 30.46°, 34.54°, 50.45°, 60.37° and 74.56° correspond to the (111), (200), (220), (311) and (400) planes of crystalline zirconia, respectively. In addition, another characteristic peak of ZrO_2_, which is indexed as (222), also appeared at 2*θ* = 62.12°, which is not clearly visible due to the low resolution of the diffractogram. Similarly, the XRD pattern of the HRG@ZrO_2_ nanocomposite in Figure 1(green line) also exhibits the characteristic XRD peaks of cubic ZrO_2_, in addition to the peaks belonging to HRG, which confirms the formation of a nanocomposite. 

### 3.2. TEM Analysis

**Figure 2:**
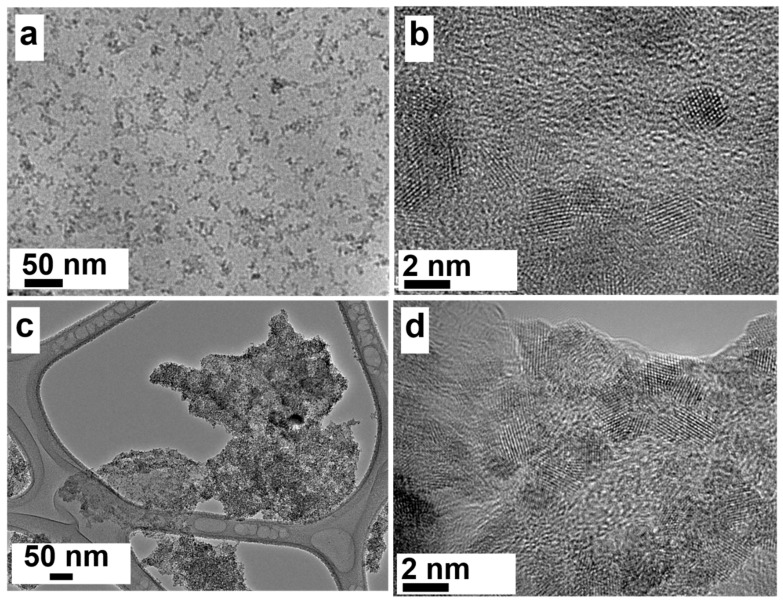
TEM images of pure ZrO_2_ NPs (**a**,**b**), and HRG@ZrO_2_ nanocomposite (**c**,**d**). Variation in the reaction conditions has led to the synthesis of ultra-small ZrO_2_ NPs.

The TEM analysis has revealed the formation of nearly spherical ZrO_2_ NPs in the size range of 1–2 nm (cf. Figure 2). It is worth noting that nanoparticles are quite monodisperse and uniform in size. The formation of well-dispersed ZrO_2_ NPs is promoted by benzyl alcohol. Indeed, a slight variation in the reaction conditions, such as temperature and amount of benzyl alcohol, compared to our previously reported method rendered much smaller sized ZrO_2_ NPs (~2 nm). On the surface of HRG, the hydroxyl groups of benzyl alcohol act as anchors and provide an excellent microenvironment for the nucleation and growth of smaller sized ZrO_2_ NPs. This results in the homogeneous coverage of ZrO_2_ nanoparticles onto the HRG surfaces. 

### 3.3. UV analysis

**Figure 3:**
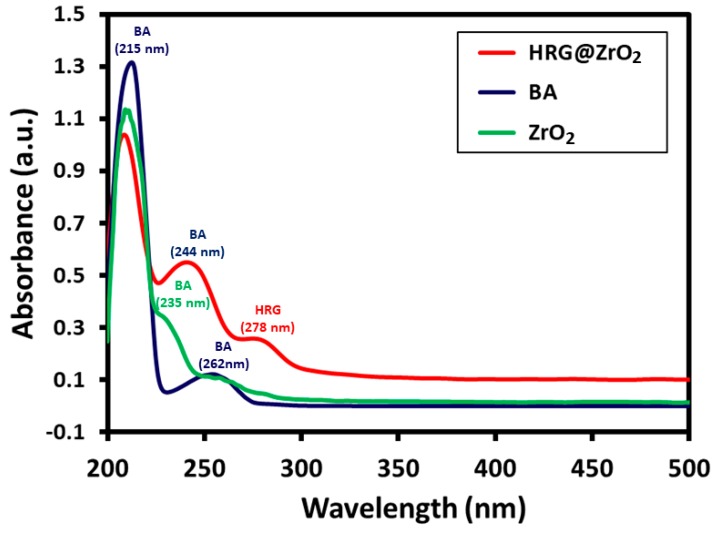
UV absorption spectra of ZrO_2_ NPs (green line) and pure benzyl alcohol (benzyl alcohol, blue line) and HRG@ZrO_2_ (red line).

**Figure 4:**
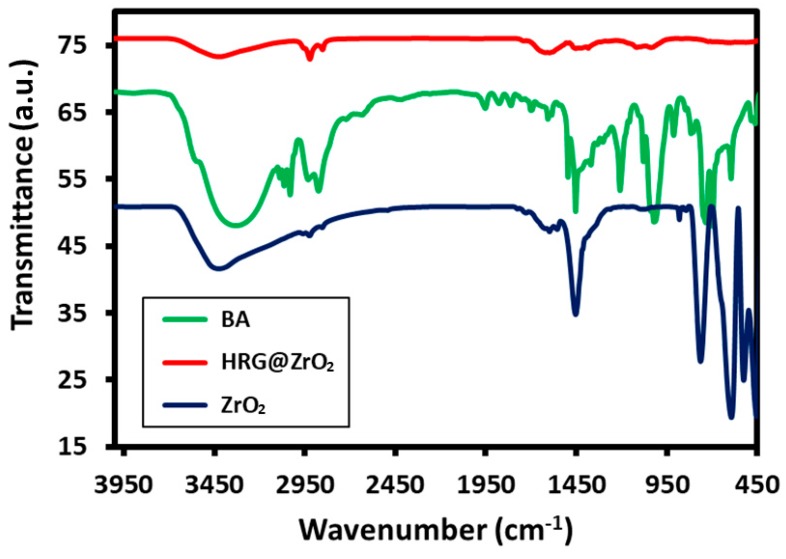
FT-IR spectra of ZrO_2_ NPs (blue line), pure benzyl alcohol (BA, green line) and HRG@ZrO_2_ (red line).

The formation as well as the stabilization of the ZrO_2_ NPs was facilitated by benzyl alcohol. The attachment of benzyl alcohol on the surface of ZrO_2_ using hydroxyl groups as anchors is confirmed by different spectroscopic techniques, including UV-Vis, FT-IR and TGA. The absorption spectrum of pure benzyl alcohol exhibits two characteristic peaks at 215 and 262 nm (Figure 3). Notably, the UV spectrum of the as-prepared ZrO_2_ clearly indicates the presence of the characteristic absorption bands of benzyl alcohol. Notably, one of the peaks of benzyl alcohol at 262 nm is slightly shifted to lower wavelength at ~235 nm in the UV spectrum of pure ZrO_2_ NPs, possibly due to interaction between benzyl alcohol and ZrO_2_. This indicates the adsorption of benzyl alcohol on the surface of ZrO_2_ NPs. Similarly, the presence of characteristic peaks of benzyl alcohol in the UV spectrum of HRG@ZrO_2_ (cf. Figure 3) also points towards the adsorption of benzyl alcohol, which may stabilize the surface of HRG@ZrO_2_ nanocomposite. FT-IR also confirmed the adsorption of benzyl alcohol on ZrO_2_ as stabilizing ligand. For this purpose, the FT-IR spectra of pure benzyl alcohol and as-prepared ZrO_2_ were measured, as shown in Figure 4. 

### 3.4. FTIR analysis

The FT-IR spectrum of the benzyl alcohol (Figure 4) exhibits absorption peaks between 3600 to 2900 cm^−1^, which represent the OH stretching of the hydrogen-bonded hydroxyl groups, C-H stretching of aromatic ring, and C−H stretching of CH_2_ (methylene) groups. The absorption peaks situated in the regions of 1900 to 1600 cm^−1^ correspond to different vibrations of phenyl rings. The peaks in the range of 1420 to 1330 cm^−1^ and 1080–1022 cm^−1^ are characteristic of O−H, and C−O stretching, respectively, of the benzyl alcohol. The cubic phase of ZrO_2_ NPs exhibits intense absorption bands in the range of 500–850 cm^−1^, which are attributed to the Zr-O bond [30]. In addition, the FTIR spectrum of the resulting ZrO_2_ also exhibits strong absorption peaks at 1460–1680 cm^−1^. These peaks disappear after calcination of the as-prepared ZrO_2_ at 600 °C for 5 h, which suggest the adsorption of organic molecules (benzyl alcohol) on the surface of nanoparticles. The comparison of the IR spectra of the as-prepared ZrO_2_ NPs, benzyl alcohol, and HRG@ZrO_2_ (cf. Figure 4) indicate the presence of benzyl alcohol in the nanocomposite. 

### 3.5. TGA Analysis

**Figure 5:**
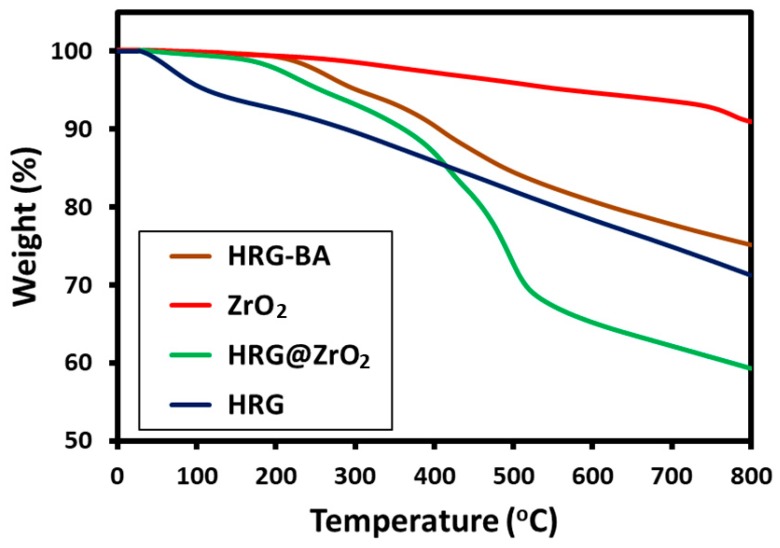
Thermogravimetric plot of (**a**) as-prepared ZrO_2_ nanoparticles (red line) (**b**) HRG@ZrO_2_ (green line), (**c**) HRG-benzyl alcohol (HRG-BA, brown line) and (**d**) HRG (blue line).

Thermal analysis was also carried out to confirm the adsorption of benzyl alcohol on the surface of ZrO_2_ NPs. Figure 5 shows the TGA peaks of pure ZrO_2,_ HRG, HRG-BA and HRG@ZrO_2_ nanocomposite. The TGA peak of pure ZrO_2_ (red line) indicates the presence of ~10 wt% of organic moieties (which are chemisorbed), including the hydroxyl groups of benzyl alcohol. Any weight loss at the temperature of lower than 300 °C is generally assigned to the removal of physically adsorbed molecules, including, methanol, water and THF etc., whereas the weight loss above this temperature is due to the desorption of chemically bonded organic moieties and the dehydration of surface hydroxyl groups. The as-prepared ZrO_2_ NPs showed a negligible weight loss of ~2% below 300 °C, while the maximum weight loss of ~8% occurred between 300 and 800 °C. This clearly indicates the interaction of hydroxyl groups of benzyl alcohol with the surface of the ZrO_2_ NPs. The TGA spectrum of pure HRG demonstrated total weight loss of ~28% in which ~10% was observed below 300 °C. The weight loss in HRG (blue line) is typically attributed to the presence of residual oxygen containing functional groups which remained on the surface of HRG, even after reduction. In order to estimate the weight loss which occurred due to the presence of benzyl alcohol, the TGA spectrum of HRG-BA (brown line) was also measured, which also demonstrated a similar weight loss to that of HRG (~25%). On the other hand, the TGA spectrum of HRG@ZrO_2_ demonstrated a higher weight loss of ~40% up to 800 °C as shown in Figure 5, green line (in this ~5% weight loss occurred below 300 °C). The higher weight loss observed in HRG@ZrO_2_ (~40%), when compared to that of pure ZrO_2_ (~10%), HRG (~28%), and HRG-BA (~25%), can be attributed not only to the degradation of the remaining oxygen containing functional groups belonging to the HRG, but also possibly due to the thermal oxidation of the resulting nanocomposite [31,32].

### 3.6. Raman and XPS Analysis

**Figure 6:**
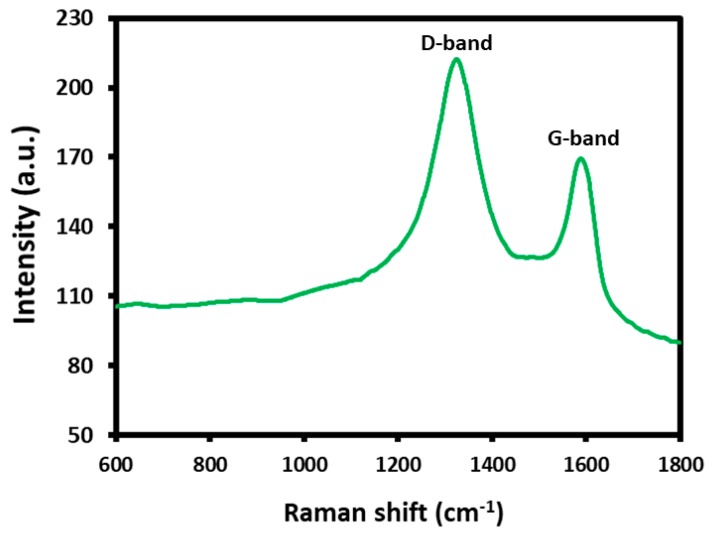
Raman spectrum of HRG@ZrO_2_ nanocomposite.

**Figure 7:**
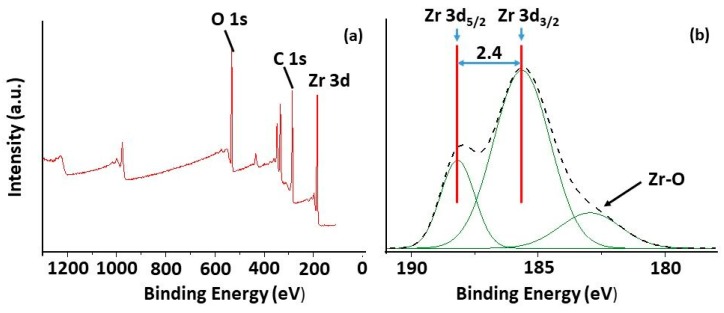
(**a**) XPS survey spectrum of HRG@ZrO_2_ nanocomposite. (**b**) The Zr 3d level XPS spectrum of HRG@ZrO_2_ nanocomposite.

The as-prepared HRG@ZrO_2_ is also characterized by Raman and XPS. The Raman spectroscopy is the most effective technique to characterize graphene-based materials. Graphene shows two Raman signals which appear around 1575 cm^−1^ and 1350 cm^−1^ and designated as G and D bands respectively. The HRG@ZrO_2_ nanocomposites showed also G and D bands of HRG centered at 1592 and 1346 cm^−1^, confirming the presence of HRG (Figure 6). The relative ratio of D/G band indicates relatively a high number of defect sites in the graphene structure. Figure 7 represents the XPS full spectrum of the HRG@ZrO_2_ nanocomposites which indicate the presence of relevant elements. The Zr 3d core level spectra (Figure 7b) show Zr 3d_5/2_ and Zr 3d_3/2_ with the peak at binding energy of 185.75 eV and 188.14 eV respectively. The energy difference of 2.4 eV between the two peaks indicates the presence of Zr^+4^ [33]. The third peak fit for the shoulder appearing at the base of Zr 3d_3/2_ could be attributed to the oxygen deficiency which could be due to the under-coordinated Zr sites of ultra-small ZrO_2_ nanoparticles [34]. 

### 3.7. Electrochemical Performance of the HRG, ZrO_2_ and HRG@ZrO_2_ Nanocomposite

Among various transition elements-based metal oxides, ZrO_2_, with its high chemical inertness and excellent thermal stability, has demonstrated excellent electrochemical properties. However, the electrochemical properties of pristine ZrO_2_ can be further enhanced by the inclusion of carbon-based nanomaterials, such as graphene. It has a large specific surface area, an extensive two-dimensional π-π conjugation structure, and possesses excellent electron conductivity which immensely contributes to enhancing the electro catalytic properties of ZrO_2_. For instance, recently, the electrochemical studies of HRG-based ZrO_2_ nanocomposite, including the Cyclic Voltammetry (CV) and Electrochemical Impedance Studies (EIS) studies, have revealed that the nanocomposite enhanced capacitance and charge transfer resistance when compared to pristine ZrO_2_ NPs [35]. Similarly, in this study we have compared the electrochemical performance of as-prepared pure ZrO_2_ NPs with those of a HRG@ZrO_2_ nanocomposite. 

**Figure 8:**
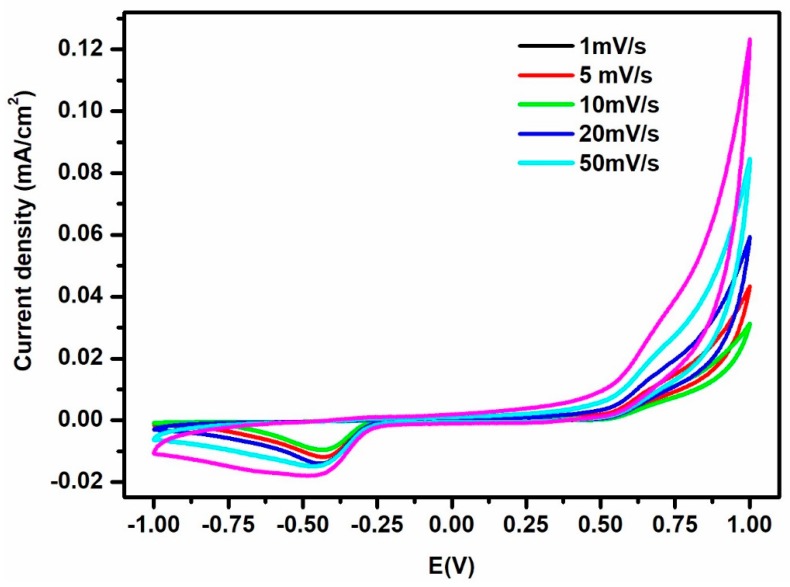
Cyclic voltammogram of HRG@ZrO_2_ nanocomposite with different sweep rate.

**Figure 9:**
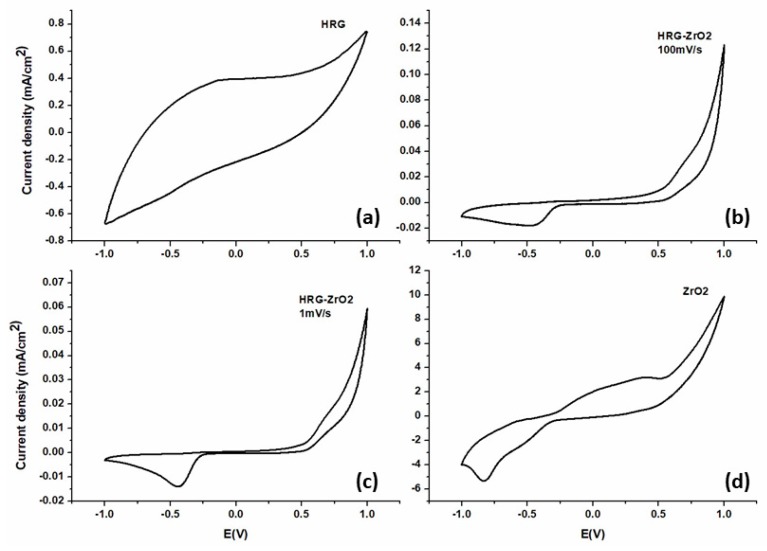
Cyclic voltammogram of ZrO_2_ and HRG@ZrO_2_ nanocomposite with different sweep rate (**a**) HRG (**b**)HRG@ZrO_2_ at 100 mV/s (**c**) HRG@ZrO_2_ at 1 mV/s and (**d**) ZrO_2_.

The Cyclic Voltammetry (CV) plots were measured within the potential region (−1.0 to 1.0 V) in 2M KOH solution. Cyclic voltammograms of HRG@ZrO_2_ nanocomposite at different sweep rates (1, 5, 10, 20, 50, mV/s) in 2 M KOH revealed interesting electrochemical behavior after the dispersion of ZrO_2_ on the surface of HRG. As shown in Figure 8, this behavior is clearly reflected in the voltammograms of the HRG@ZrO_2_ nanocomposite electrode, which were recorded in N_2_ saturated 2M KOH solution at different scan rates. The capacitance of the material increases linearly with increasing the scanning rate. For the purpose of comparison, the capacitance of pure ZrO_2_ NPs, pristine HRG and a HRG@ZrO_2_ nanocomposite electrode was also measured, as shown in Figure 9. Notably, no redox peaks were observed in the CV of pure HRG, and the absence of redox peaks in HRG is attributed to the presence of various oxygen containing functional on the surface of HRG. However, upon the dispersion of ZrO_2_ on HRG, the voltammogram of HRG@ZrO_2_ clearly shows a sharp reduction peak at −0.445 V with respect to Ag/AgCl electrode at a lower scanning speed of 1 mV/s. This peaks broadened and slightly shifted to −0.465 at a scanning rate of 50 mV/s. The slight change in the shape of the redox peaks at different scanning speeds indicates the conductivity of the material in 2 M KOH solution. The voltammogram of pure ZrO_2_ also demonstrates a sharp reduction peak at −0.827 V at a high scanning rate. The relatively broad redox peak in HRG@ZrO_2_ at high scanning rate when compare to pure ZrO_2_ clearly indicates the better capacitance of the nanocomposite [35]. The increase in capacitance shows that the electron transfer process occurring at HRG@ZrO_2_ nanocomposite electrode is a surface-confined process. Therefore, these types of materials can be efficiently used is various sensing applications.

**Figure 10:**
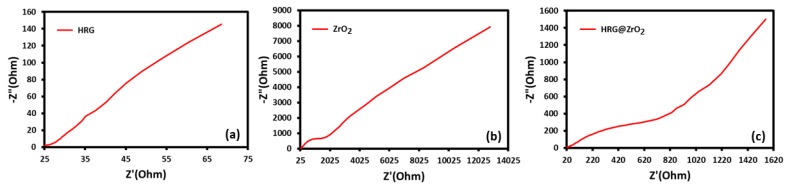
Electrochemical impedance spectroscopy plots of HRG, ZrO_2_ and HRG@ZrO_2_. (**a**) HRG (**b**) ZrO_2_ (**c**) HRG@ZrO_2_.

Electrochemical impedance spectroscopy (EIS) was carried out using a frequency range of 10^5^ to 10^−1^ Hz in 2 M KOH, using three electrode cells connected to an electrochemical station. In this system, the electrodes prepared from the aforementioned samples (HRG, ZrO_2_ and HRG@ZrO_2_) served as the working electrode, a platinum electrode as counter, and a silver electrode as a reference. The EIS technique is an effective electrochemical method which typically demonstrates the electron transfer phenomenon across the sample and electrolyte interface. Figure 10 exhibits the Nyquist plots of HRG, ZrO_2_ and HRG@ZrO_2_ electrodes, consisting of real part Z′ of impedance on x-axis and inverse of imaginary part −Z″ on y-axis. The measured electrodes have demonstrated different behavior on the Nyquist plots. The ZrO_2_ demonstrates a slight semicircular curve in the high frequency zone and an inclined straight line in the low frequency region, which represent a typical AC impedance curve for capacitance. In contrast, in the case of HRG, only inclined straight lines were observed in all regions. However, after the dispersion of ZrO_2_ on the surface of HRG (HRG@ZrO_2_), the slight semicircle part in the high frequency zone increases compared to pure ZrO_2_. The diameter of the semicircle in the high frequency region represents the charge transfer resistance, which is generally associated with the pore structure of materials. Similarly, in a recently published study, the diameter for a HRG-based ZrO_2_ nanocomposite was also found to be lower, indicating a charge transfer resistance of the material [36]. The Warburg impedance (inclined straight line), which is generally characterized by having real and imaginary identical contributions of impedance, leads to the formation of a phase angle of 45°, which is responsible for the generation of a line in the impedance plot.

## 4. Conclusions

Herein, we present a one-pot solvothermal approach for the preparation of stabilized cubic ZrO_2_ nanoparticles and HRG@ZrO_2_ nanocomposite using benzyl alcohol both as solvent and a stabilizing agent. The resulting ZrO_2_ NPs were characterized by XRD, TEM, and TGA. During the synthesis, the temperature and amount of benzyl alcohol have a significant effect on the size of resultant NPs. Slight variation in the temperature resulted in the formation of ultra-small, cubic ZrO_2_ NPs with an average diameter of 1–2 nm. In the case of HRG@ZrO_2_, these smaller-sized NPs uniformly coat the surface of HRG. The adsorption of benzyl alcohol on the surface of ZrO_2_ NPs and its role as a stabilizing agent was confirmed with UV-Vis and FT-IR spectroscopies. It is revealed that the OH groups of benzyl alcohol facilitated the nucleation of ZrO_2_ NPs by providing an excellent microenvironment. The HRG@ZrO_2_ nanocomposite has demonstrated enhanced electrochemical properties after the dispersion of ZrO_2_ NPs. The as-synthesized HRG@ZrO_2_ nanocomposites resulted in improved electrical communication, which could be interesting to explore in sensing applications.
